# Uterine leiomyoma: understanding the impact of symptoms on womens’ lives

**DOI:** 10.1186/1742-4755-11-10

**Published:** 2014-01-30

**Authors:** Luiz Gustavo Oliveira Brito, Marislei Sanches Panobianco, Maurício Mesquita Sabino-de-Freitas, Hermes de Freitas Barbosa, George Dantas de Azevedo, Luciane Maria Oliveira Brito, Francisco José Candido-dos-Reis

**Affiliations:** 1 Department of Gynecology and Obstetrics, Ribeirão Preto School of Medicine, University of São Paulo, Avenida Bandeirantes, 3900, 8th floor, Monte Alegre, Ribeirão Preto, SP, Brazil; 2 Ribeirão Preto College of Nursing, University of São Paulo, Ribeirão Preto, Brazil; 3 Department of Pathology, Ribeirão Preto School of Medicine, University of São Paulo, Ribeirão Preto, Brazil; 4 Federal University of Rio Grande do Norte, Natal, Brazil; 5 Federal University of Maranhão, São Luis, Brazil

**Keywords:** Uterine leiomyoma, Health related quality of life, Counseling, Qualitative research, Abnormal uterine bleeding, Pelvic pain

## Abstract

**Background:**

Most women report negative experience about the symptoms of uterine leiomyoma (UL) in their lives, such as abnormal uterine bleeding and pelvic pain. Many studies have been conducted about efficacy of UL treatment, but little research has been performed about womens health related quality of life (HRQL).

**Methods:**

This is a semi-structured, descriptive, observational, qualitative study that was performed during eight months. Focus group (FG) interviews were performed with women attending at a tertiary hospital in Brazil, who were consecutively included in the study. Seventy women with symptomatic UL were recruited to this study. FG duration was one hour with mediators with 5-6 women at each group. Collected data from discussions was processed according to thematic analysis and stored at a qualitative software.

**Results:**

Women were negatively influenced by the presence of symptomatic UL. The major themes that were noticed during analysis were: beliefs and attitudes towards UL; limitation to social and professional activities; sensation of fear/unfairness/discouragement towards the symptoms and adverse effects during treatment with GnRH analogs.

**Conclusions:**

Symptomatic UL has a negative impact on womens HRQL. Health providers should consider such impact when counseling women on their treatment options, since it may have an important influence in these patients’ decision-making process. While current pharmacological treatments may improve disease specific outcomes, such as bleeding intensity and tumor volume, they fail on actually improving quality of life.

## Background

Uterine leiomyoma (UL) is the most prevalent benign gynecological tumor. From 100 women, 80 present this tumor; however, only around 30% will report symptoms [1]. Treatment of this condition has undergone a significant transformation in recent years [2]. Novel and more efficient drugs have been developed to treat symptomatic UL; however, surgical procedures are still highly prevalent in some countries to treat UL. In the United States, UL are the main reason for performing hysterectomies [3] at an annual expense of 2 billion dollars [4].

The pharmacological approach for UL involves hormonal and nonhormonal treatments. Progesterones generally reduce uterine bleeding that is caused by the tumors. GnRH analogs are FDA-approved for UL and block the hypothalamic-pituitary-ovarian hormonal axis, causing a hypoestrogenic effect that leads to a reduction in uterine fibroid volume. Nonhormonal treatments, involving antifibrinolytics and nonsteroidal anti-inflammatory drugs (NSAIDs), are also used [5].

Before considering hysterectomy, minimally invasive procedures for the treatment of UL can be offered, such as surgical hysteroscopy, uterine artery embolization (UAE), uterine artery occlusion, high-intensity focused ultrasound, cryomyolysis, and laparoscopic myomectomy [5]. However, factors limiting the use of these procedures include their high cost and the fact that not all of them are provided by some public health systems, such as in Brazil.

It is known that UL causes abnormal uterine bleeding and pelvic pain as main symptoms, impairing womens’ health related quality of life (HRQL). However, this last parameter was the object of fewer researches [6]. Until this moment, there is only one condition-specific questionnaire for UL, the Uterine Fibroid Symptom and Quality of Life (UFS-QOL) [7]. Qualitative studies are important to explore womens’ feelings about the impact of UL in their lives, the barriers or problems related to pharmacological treatment of UL and to understand their motivations for requesting clinical and/or surgical treatment. Accordingly, we aimed to perform an exploratory study with focus groups addressing specific information about their personal impression of UL.

## Methods

This study was conducted at our Department’s Urogynecology and Gynecological/Pelvic Reconstructive Division (HCFMRP-USP), which was created 20 years ago and provides care for women with benign gynecological diseases and urogenital disorders. Our tertiary center receives patients from all countryside cities of São Paulo state. This observation motivated us to study womens’views on UL. The study concept and its objectives were explained to the patients during their consultation. The patients were consecutively included in the study, and inclusion took place for a period of eight months. Women who accepted to participate in the study were assigned to a focus group (FG) discussion session, after signing an informed consent form. Our Institutional Review Board Comitê de Ética em Pesquisa do HCFMRP-USP approved the study. We excluded women that had already decided with his/her physician which treatment she would do, or patients who had some form of emotional/psychological instability that prevented them from taking part in the discussions (n = 8).

The FG sessions [8] took place in a quiet space near the outpatient clinic, where we had chairs arranged in a circle. Two male gynecologists (L.G.O.B; P.S.M.) were involved in the sessions: one served as mediator and the other as an observer. The discussions were held according to a guiding script and were divided into three parts. Four to six women were included at each group, with 70 participating on total; we did not have any refusal from the patients. Some patients from the group had reported that they had already tried nonsurgical treatments. All of them had been followed at our facility for at least six months prior to the occurrence of FGs. This was thought to be necessary because some patients were not treated adequately before referral to our hospital according to available guidelines for the treatment of UL.

We initially presented the study, the purpose of the discussion, and the contributions the group could make towards the development of a better UL treatment. After the introduction, the patients were given time to express any uncertainties they had about the study. Then, the main issues were approached through some questions, such as:

• What is your understanding of uterine leiomyoma (uterine fibroids) disease?

• How has this condition affected your life?

• What are your treatment expectations?

We concluded with a summary of the discussion, clarifying some misconceptions that participants might have had about the disease. None of the patients attended more than one FG discussion; content analysis was performed using a single time-point. Since the participants were still being evaluated, and therefore, not all of them had undergone clinical treatment or decided on the type of treatment they wanted. The median duration time for group discussions was 60 minutes, ranging from 30 to 93 minutes.

Each participant was issued an identification code. FGs were recorded (*Audacity version 1.3.12, Free Software Foundation, United States*) and then transcribed verbatim to ensure that all ideas retained their original form. The observer transcribed the recordings immediately after each FG discussion to prevent loss of details. Transcribed statements were analyzed and coded using an additional software (*Atlas Ti version 6.2, GmBH, Berlin, Germany*). Transcriptions were read by two persons (L.G.O.B, F.J.C.R) and agreed on the themes identified.

We used content analysis [9], which identifies the main themes and subthemes raised during the FG discussions and the different opinions about them. The saturation criterion was applied, such that data collection was terminated when comments containing new content and meaning could no longer be identified. Diverse content categories were established within the themes prepared by the researchers. Finally, after preparing the themes, two researchers (M.S.P, G.D.A) reviewed them.

## Results

Table 1 comprised all sociodemographic data from the studied patients’. Women discussed the impact of UL and discussion categories were built, leading to four recognizable major themes, which were: knowledge (beliefs and attitudes) towards UL; fear, unfairness and discouragement; changes in social and professional activities due to bleeding and pelvic pain; and medications for UL.

**Table 1:** Baseline characteristics of participants

**Groups variable**	**N (%)**
Age (median, years)	70 (31-53)
**Marital status**	
Without partner	18 (25.7%)
With partner	52 (74.3%)
**Parity**	
0	6 (8.6%)
1	6 (8.6%)
2+	58 (82.7%)
**Education (years)**	
< 5	15 (21.4%)
5-11	24 (34.3%)
>11	31 (44.3%)
**Occupation**	
Employed	61 (87.1%)
Unnemployed	19 (12.9%)
**Family income** (1 minimum wage = 300 dollars)	
1	9 (12.8%)
2-4	42 (60%)
5+	19 (27.2%)
**Comorbidities**	
Yes	39 (55.7%)
No	31 (44.3%)
**Total**	**70 (100%)**

### Beliefs and attitudes toward uterine leiomyoma

Our study showed that participants were concerned that uterine leiomyoma could develop into cancer. Their fear of such an outcome was a motivational factor for seeking clinical and/or surgical assistance, and was obvious from their facial expressions as they discussed the disease. The misconception of uterine leiomyoma as a preneoplastic disease of uterine cancer was a justification to undergo hysterectomy. While several participants had relatives with gynecological malignant neoplasms, others also reported acquiring this information from family members, their partner, or acquaintances, rather than directly from a woman who had experienced this type of cancer.

*Look, I do not know much about myoma, but I know that we cannot allow much time to go by without treating it, since it can then lead to another disease…Cancer, isn’t it? We worry thinking about it because my family, my mother’s family, all had cancer. My mother had myoma; she did not seek care, and then developed into cancer.* (W18, 52 years)

Another group of participants reported having little information about leiomyoma. Some of these women were dissatisfied with their lack of knowledge about the disease and sought out information about it. Physicians and media (internet and television) were their main sources of information. The discussion indicated that the women believed their physicians were responsible for providing them with knowledge.

*I don’t know much about it, only what the doctor tells us, and then you get worried because it is just what you heard, you know?* (W15, 42 years)

### Fear, unfairness and discouragement

Women referred that bleeding and pelvic pain caused a huge sensation of fear, especially because of the unpredictability of these symptoms. Most patients had already tried pharmacological treatment and experienced therapeutic failures, which made them feel discouraged; they felt they did not have enough strength to keep on trying clinical treatment. Unfairness was another feeling reported by women; they could not understand how they used medications correctly and they did not work.

“…*I feel a lot of pain, a lot of pain…When it comes, I sweat a lot [and] am cold…and it causes too much pain. […] I spent one month and 20 days having nonstop menses. The only medication that blocked the bleeding was subcutaneous, but today I will take the last dose that I can take and then, I am going to do what? It’s going to start bleeding again? And the cramps? It feels like I am having a baby! It causes strong contractions for a week. So…I don’t think it is fair…* (W8, 47 years)

*“…I am starting to bleed again, and I am scared!”* (W11, 36 years)

*“…I sleep every night with that baby diaper on me…I cannot go out because blood falls under my panties, it goes out and spills a lot, you know…Because tampon can not control it…”* (W16, 39 years)

### Changes in social and professional activities due to bleeding and pelvic pain

The participants reported that bleeding and pelvic pain limited their domestic and social activities. Some women reported going to hospitals after many episodes of uterine bleeding and pelvic pain. Professional activity was also impaired by symptoms. Many women used their symptoms to justify their decision to undergo hysterectomy.

*“I brought my sister to the hospital for an eye surgery, and blood started to come out and my pants were all with blood, I needed three tampons to stop, I was ashamed at the hospital”.* (W20, 37 years)

*“I am also feeling the same things. Bleeding is the worst thing in the world. I have suffered a lot…I am praying to God that I can have surgery because I cannot stand it anymore. I have lost too much blood, and I have spoken to a physician. I couldn’t walk properly at home, so I went to the hospital and they told me that I should go to a blood bank because I was too anemic for surgery....I turned on the shower, and I saw those large blood clots going down the drain…and the drain got stuck. So I called my husband, and he called an ambulance, because it seemed that I was fainting*”. (W10, 48 years)

### Adverse effects using GnRH analogs

Most medication-related complaints were associated with the use of goserelin, which had a negative impact on the patients’ lives. Such medication was easily recognized by these women due to its subcutaneous route of administration. Despite its beneficial effects on uterine bleeding and pelvic pain, in general, most women reported that their quality of life considerably worsened after using this drug; they reported unpleasant vasomotor and urogenital symptoms usually associated with hypoestrogenism. Add-back therapy was offered to these patients; however, only few patients could actually use the medication due to financial limitations.

*The injection gave me some hot flashes, feelings of fear, [as well as] a bad feeling… sometimes I could not sleep. I could not work because of the unbearable heat; then I started to pass out. This hot feeling came from the injection because I didn’t have… When you lay down on the bed, your body stays hot, totally covered in sweat…on the blanket…It’s horrible—completely absurd… *(W8, 47 years, using GnRH analogs)

*When this hot feeling caused by the treatment comes to me, and I am sleeping, I have to take off the blanket… Sometimes, I run to the backyard and take a hose bath there, not even in the shower [because] the hose is colder… Then, my family asks, ‘mom, what the hell is going on…?’ However, once I get wet I feel refreshed… Another thing that I noticed is that my vagina is very dry, and when urine is released it touches the vagina, [and] that hurts a lot. I did not have this… *(W10, 48 years, using GnRH analogs)

## Discussion

This study has found that women with uterine leiomyoma had a negative impact in their quality of life when the symptoms had started, and treatment failure with medications triggered diverse negative sensations and modified coping strategies towards this disease. GnRH analogs caused a quick recovery from UL symptoms; however, due to menopausal symptoms, women reported that their quality of life had worsened.

The wrongful association of uterine leiomyoma with cancer was widely reported by patients during the interviews. However, we could not find any uterine leiomyoma study discussing the effects of cancerophobia. In fact, the risk for leiomyosarcoma is extremely low, and this tumor is often incidentally detected in hysterectomy specimens [10]. Moreover, lack of knowledge of the actual physiopathology of the disease may influence the treatment decision-making process; therefore, it is worrisome that women do not have adequate knowledge to make the right decision. A study conducted in the United States divided 300 women who were scheduled to undergo hysterectomy to treat UL into 2 groups: the first group received monthly leaflets, a DVD, and a consent form about the surgery, and the second group was given the chance to attend a consultation with a health professional about the disease. Two months later, a questionnaire showed that women in both groups had more knowledge of the disease and expressed satisfaction with their decision to undergo hysterectomy; however, their treatment decisions and preferences remained the same as before [11].

Uterine leiomyoma symptoms impaired significantly womens’ lives. It was shown during interviews that uterine bleeding lead to negative sensations, such as fear and unfairness.. These feelings impair the social and health world of women who suffer with this disease. They are considerably discouraged to keep on trying conservative treatment. This is reinforced with a *bridged pseudopathway* of UL toward uterine cancer as well as with the concept that women should not worry about having a hysterectomy after completing childbearing. However, some studies showed that one of the main symptoms, vaginal bleeding, might be ultimately overestimated. Nevertheless, physicians do inquire about the severity of vaginal bleeding during diagnosis.

Previous studies have demonstrated that the intensity with which women complain about vaginal bleeding is the same, whether or not they are aware that they have UL [12]. An exploratory study using an Interpretative Phenomenological Analysis (IPA) approach found that women use multiple experiences and pieces of information to understand their conditions, treatment options, and future health expectations [13]. Individual differences in disease representation may correspond to differences in womens’ relative stress levels. This indicates the importance of identifying other medical and psychological concerns of women diagnosed with this disease.

It is accepted that only large or pedunculated leiomyomas can cause the pelvic pain symptom. However, some women, especially those who are used to comparing the size of a tumor to an object, complain of pelvic pain. Therefore, health professionals who counsel patients with uterine leiomyomata should explain that pelvic pain is not always related to the presence of such tumors, but may be associated to other pathologies, such as adenomyosis.

This qualitative research has analyzed the patients’ satisfaction after using GnRH analogs. Satisfaction with these drugs was also investigated with other benign gynecological diseases, such as endometriosis; a previous study found that GnRH analogs did not lead to chronic pain relief [14]. Initially, we did not include the use of goserelin acetate in the present study design; however, during FG discussions, we recognized the importance of discussing about the use of goserelin because of its negative effect on womens’ HRQL. This observation should be considered when prescribing such medications.

However, one of this study’s limitations was that we were unable to longitudinally analyze our participants’ qualitative comments. We propose that it would be useful for future qualitative studies to analyze the patients’ perceptions about their disease and the proposed treatment over time. We also did not use any specific questionnaire for fibroids since the only one available (UFS-QOL) was not validated for Portuguese language.

We hope it contributes to the understanding of patients’ experiences with uterine leiomyoma, especially among health professionals who treat this condition. Health professionals need to comprehend individual and cultural differences contributing to a woman’s understanding of her condition. Quality of information is important to the patient and to all who surround her [15]. Treatment of this pathology is multidisciplinary (involving psychology, nursing, and many other fields). The doctor-patient relationship, in addition to other motivational factors, should be considered during the patient’s decision-making process. Moreover, verbal and nonverbal communications from the patients should be considered. We noted during our study that the perception of care seemed quite different between patients and clinicians, and both sides should better comprehend this difference. This approach would help patients understand why it can be difficult to choose a treatment, and physicians comprehend how different influences on individual women can delay them from reaching a final decision on a specific treatment option.

## Competing interests

The authors declare that they have no competing interests.

## Authors’ contributions

LGOB conceived and designed the study, acquired and analyzed the data and drafted/revised the article; FJCR conceived and designed the study, analyzed the data and revised the article; MSP, GDA, HFB, LMOB participated in analyzing data and revising the article; MMSF participated in revising the article. All authors approved the final version of the article.
